# What are the key factors contributing to the inequity in healthcare resource allocation? Evidence from China’s health panel data from 2009 to 2021

**DOI:** 10.3389/fpubh.2025.1586585

**Published:** 2025-07-18

**Authors:** Enhong Dong, Tingting Wang, Ting Xu, Xueting Chen, Qianqian Zhou, Weimin Gao, Yuping Liu

**Affiliations:** ^1^School of Nursing and Health Management, Shanghai University of Medicine & Health Science, Shanghai, China; ^2^Institute of Healthy Yangtze River Delta, Shanghai Jiao Tong University, Shanghai, Shanghai, China; ^3^School of Nursing, Kunming Medical University, Kunming, China; ^4^School of Humanities and Management, Kunming Medical University, Kunming, China

**Keywords:** inequity, health-care resources, concentration index, determinants, China

## Abstract

**Background:**

As economic growth drives higher demand for health services, equitable health resource allocation becomes crucial to meet diverse healthcare needs. Since China’s reform and opening-up, increased government healthcare investment has not fully resolved regional disparities. Existing studies, often relying on methods other than the concentration index, fail to comprehensively analyze the link between resource inequities and economic factors. This study uses the concentration index and its decomposition to assess regional disparities and identify determinants of inequity, offering practical recommendations for optimizing resource distribution in China and similar developing nations.

**Methods:**

This study analyzed China’s healthcare resource allocation (institutions, beds, and workforce) from 2009 to 2021 using the concentration index to measure equity across socio-economic regions and its decomposition method to identify contributing factors to inequality.

**Results:**

From 2009 to 2021, the numbers of institutions per 1,000 people (IPK), beds per 1,000 people (BPK), doctors per 1,000 people (DPK), technicians per 1,000 people (TPK), and nurses per 1,000 people(NPK) in China increased. The concentration index (CI) for IPK remained negative, while BPK’s CI turned negative after 2013. CIs for DPK, TPK, and NPK stayed positive. The CI for IPK’s absolute value rose, while others decreased. Factors like population size (PS), population density(PD), geographical Location(GL), maternal mortality rate(MMR), rate of born-baby weighting less than 2.5 kg (RBWL25), and perinatal mortality rate (PMR) influenced unequal healthcare resource distribution, with PS and RBWL25 favoring developed areas, and PD, GL, and MMR favoring less developed regions. Additionally, urbanization level (UL), Out-of-Pocket (OPP), per capita health expenditures(PCHE), per capita gross domestic product(PCGDP), disposable income of urban residents(DIUR), government health expenditures (GHE), and number of insured(NI) positively impacted resource allocation to developed provinces, with varying effects.

**Conclusion:**

This study analyzes 2009–2021 panel data, revealing growth trends and regional disparities in China’s healthcare resource equity, focusing on institutions, beds, and workforce. Need variables (PS, PD, and RBWL25) reduced bed/doctor disparities, while MMR/PMR worsened maternal/nurse inequities. Non-need economic factors concentrated resources in affluent areas despite redistribution efforts. The findings highlight ongoing challenges in equitable distribution and offer crucial policy insights for China and other developing nations.

## 1 Introduction

With the continuous development of the economy, people’s demand for health services is showing trends of increasing, multi-level, and diversified. However, health resources are scarce resources, and in order to meet the increasingly diverse health service needs of people as much as possible, rational allocation of resources is the prerequisite foundation (1). To our best of knowledge, the equity of health resource allocation is a crucial objective of public health policy and health system management, as it directly impacts the overall health status and social welfare of society (2). Achieving equity in health resource allocation necessitates collaborative efforts from the government, social organizations, and the public. Acknowledging the critical significance of health resource allocation, the Chinese government has actively sought to enhance investment in healthcare since initiation of reform and opening-up policies. This has involved the expansion of hospital facilities, an increase in the numbers of available beds, and the recruitment of additional medical personnel, including doctors and nurses, in order to address the escalating demand for medical resources among the population. In conjunction with augmenting supply-side investments, reforms have also been instituted on the demand side, which include initiatives aimed at increasing the number of insured individuals, elevating the urbanization rate, and boosting the disposable income of urban residents as well as the net income of rural farmers, among other strategies. This trend of heightened investment persisted until the implementation of the new healthcare reform in 2009, which yielded substantial phased advancements in China’s healthcare system, effectively mitigating the challenges of accessibility and affordability of medical care for the public. However, China’s extensive geographical expanse and inherent diversity, coupled with uneven development, a wide array of economic activities, population growth, cultural variances, geographical factors, and transportation conditions across different regions, as well as the fragmentation of the urban–rural dual system, have contributed to the unequal distribution of healthcare resources (3, 4). Notable disparities in the allocation of healthcare resources can be observed both between and within various regions of China (5, 6).

Indeed, China’s healthcare system reflects a complex interplay of historical legacies and reform-driven changes. Established under a planned economy, it initially provided universal but basic coverage through state-owned enterprises (urban) and the Cooperative Medical System (rural). Economic reforms post-1978 dismantled rural collectives, collapsing the rural CMS and exacerbating urban–rural disparities. The 2009 healthcare reform sought to reverse this fragmentation by expanding insurance coverage (e.g., NCMS for rural residents), investing in primary care, and reducing out-of-pocket costs. Yet, structural inequities persist: urban areas concentrate majority of tertiary hospitals, while rural regions face workforce shortages and underfunded infrastructure. Regional disparities are further amplified by uneven economic development, with eastern provinces outperforming central/western areas in bed density and physician ratios. The urban–rural dual system, hierarchical diagnosis gaps, and the “siphon effect” of talent migration to cities remain unresolved, despite policies like the “Western Development Strategy” (7, 8).

In response, the Chinese government has enacted policies aimed at optimizing the distribution of healthcare resources to promote equitable access to healthcare for its citizens. Since the initiation of the new medical reform in 2009, the government has significantly increased its investment in healthcare resources, with a particular emphasis on providing financial support to the central and western regions. This has included the expansion of medical insurance coverage, enhancement of primary healthcare services, establishment of a hierarchical diagnosis and treatment system, and efforts to reduce per capita health expenditures as well as disparities in healthcare resource allocation between developed and underdeveloped regions (1).

In the context of research examining the inequities in the allocation of healthcare resources and the factors that influence these disparities, Horev et al. identified that variations in socioeconomic factors play a significant role in generating income inequality (9). This income inequality, in turn, results in unequal investments in both employment and health, which ultimately affects the fair distribution of health resources. Marmot identified nine principal social determinants that contribute to health service inequality, emphasizing that the factors driving poverty across nations are paramount in exacerbating health service inequity (10). Kreng and Yang employed grey relational analysis to examine the equity of health resource distribution in Taiwan, revealing that the disproportionate allocation of health resources in northern Taiwan resulted in significant regional disparities, characterized by a concentration of large hospitals in urban areas and limited access to health services in rural regions (11). They contended that the unilateral payment system of the National Health Insurance (NHI) plays a crucial role in enhancing the fairness of health services. Wang et al. conducted a comparative analysis of health service utilization (both outpatient and inpatient) among middle-aged and older adult populations in Gansu and Zhejiang provinces, finding that both regions exhibited inequitable health service utilization favoring wealthier individuals, with the disparity being more pronounced in the wealthier province (12). They identified income differences as the primary determinant, while health insurance coverage, service provision modalities, and the proportion of out-of-pocket payments also contributed to the inequity of healthcare resources. Yang Lin, Cheng Qian, and Li Yuan utilized a state-space model to assess the time-varying elasticity of various health investments on the urban–rural health resource gap in China from 1985 to 2011, concluding that household health expenditure was instrumental in reducing the urban–rural health resource allocation gap. They noted that the elasticity of different health expenditure proportions fluctuated prior to 2002 and stabilized thereafter (13). Xie posited that, in addition to income inequality, the types of medical insurance available to urban residents are also significant determinants of health service equity (14). Li observed a trend of wealth concentration in the utilization of maternal health services in rural western China, which correlates with income levels, educational attainment, and geographical accessibility (15). Xia Yuqi et al. argued that environmental factors, including economic development, population density, policy support, social infrastructure, and institutional operations, significantly influence the inequity of health resource allocation (16). Ye Yizhong and Tao Qunshan found that regional GDP, resident population, and urbanization levels have a substantial impact on the inequity of health resource allocation (17). Xu Xinrui et al. contended that regional GDP and per capita disposable income positively affect the mitigation of healthcare resource inequity, while government health expenditure and the proportion of the population aged 65 and above negatively influence the equity of healthcare resource allocation (18).

Currently, scholars primarily utilize analytical techniques such as the coefficient of variation (19), Gini coefficient (3, 20), and Theil index (6, 21–24) to assess disparities in the allocation of health resources. However, these approaches do not adequately capture the intricate relationship between inequities in healthcare resource distribution and factors related to economic development. In contrast, the concentration index method provides a more effective framework for illustrating the distributional patterns of health resources across different counties or regions, and it may reveal correlations between healthcare resource allocation inequities and economic development factors, often relying on macro-level datasets or cross-sectional data (25). On the other hand, the concentration index decomposition method is predominantly employed in research utilizing microdata, concentrating on individual-level health inequities (14, 26).

Consequently, this research utilized panel data spanning from 2009 to 2021, sourced from the China Statistical Yearbook and the China Health Statistical Yearbook covering the years 2010 to 2022. The study employed a comprehensive methodology that integrates the concentration index and its decomposition to examine the regional disparities and determinants of inequity in health resource allocation in China during the specified period. The findings of this study may offer practical recommendations for governmental policy formulation and optimization regarding health resource allocation, as well as assist in the strategic planning of regional health service distribution, thereby addressing the inequities in healthcare resource allocation in China and other developing nations exhibiting similar characteristics.

## 2 Materials and methods

### 2.1 Data sources

#### 2.1.1 Data source and classification

This study used data from the “China Health Statistics Yearbook” and “China Statistical Yearbook” of 31 provinces (municipalities or autonomous regions) from 2010 to 2022. The data on health resources and human resources, including outcome variables [institutions per 1,000 people (IPK), beds per 1,000 people (BPK), the numbers of doctors per 1,000 people (DPK), technicians per 1,000 people (TPK), nurses per 1,000 people (NPK)], need variables [population size (PS), population density (PD), maternal mortality rate (MMR), rate of born-baby weight less than 2. 5 kg (RBWL25), perinatal mortality rate (PMR)], and non-need variables [per capita gross domestic product (PCGDP), urbanization level (UL), net farmers ‘income (NFI), disposable income of urban residents (DIUR), number of college students (NCS), numbers of insured (NI), per capita health expenses (PCHE), out-pocket payment (OPP), government health expenditures (GHE), geographical location (GL)]. The variables and their descriptive characteristic were present in Table 1.

**Table 1:** Variables and their description in the study.

Variables	Description	Unit	Data type	Data resource
Outcome variables				
IPK	Institutions per 1,000 people	None	Continuous	China Health Statistics Yearbook
BPK	Beds per 1,000 people	None	Continuous	China Health Statistics Yearbook
DPK	Doctors per 1,000 people	None	Continuous	China Health Statistics Yearbook
TPK	Technicians per 1,000 people	None	Continuous	China Health Statistics Yearbook
NPK	Nurses per 1,000 people	None	Continuous	China Health Statistics Yearbook
Need variables				
PS	Population size	None	Continuous	China Statistical Yearbook
PD	Population density	None	Continuous	China Statistical Yearbook
MMR	The annual number of female deaths per 100,000 live births	Persons per 100,000 live births	Continuous	China Health Statistics Yearbook
RBWL25	The annual number of live-born infants weighting less than 2,500 grams per 100 live births	%	Continuous	China Health Statistics Yearbook
PMR	The annual number of stillbirths and deaths in the first week of life per 1,000 total births	‰	Continuous	China Health Statistics Yearbook
Non-need variables				
PCGDP	*Per Capita* Gross Domestic Product	Yuan	Continuous	China Statistical Yearbook
UL	Urbanization Level	%	Continuous	China Statistical Yearbook
NFI	Net Farmers’ Income	Yuan	Continuous	China Statistical Yearbook
DIUR	Disposable Income of Urban Residents	Yuan	Continuous	China Statistical Yearbook
NCS	Number of College Students	None	Continuous	China Statistical Yearbook
NI	The number of Insured population	10,000 persons	Continuous	China Statistical Yearbook
PCHE	*Per Capita* Health Expenditures	None	Continuous	China Statistical Yearbook
OPP	Out-of-Pocket Expenditure on health	None	Continuous	China Statistical Yearbook
GHE	Annual Government Health Expenditures	None	Continuous	China Statistical Yearbook
GL	Geographical Location	None	Categorical	China Statistical Yearbook

IPK, institutions per 1,000 people; BPK, beds per 1,000 people; DPK, doctors per 1,000 people; TPK, technicians per 1,000 people; NPK, nurses per 1,000 people; PS, population size; PD, population density; MMR, maternal mortality rate; RBWL25, rate of born-baby weighting less than 2.5 kg; PMR, perinatal mortality rate; GHE, government health expenditures; OOP, Out-of-Pocket Payment; PCHE, per capita health expenditures; PCGDP, per capita gross domestic product; NI, number of insured; NCS, number of college students; DIUR, disposable income of urban residents; NFI, Net Farmers’ Income; GL, geographical location; UL, urbanization level.

Notably, MMR, RBWL25, and PMR are widely recognized indicators of healthcare needs in epidemiological and health policy literature (e.g., WHO benchmarks). They reflect population-level health risks that necessitate resource allocation (e.g., areas with high MMR require more maternal health services). These variables were selected to capture health needs distinct from socioeconomic status (SES), aligning with studies like Victora CG et al. and Enhong D. et al., which use health outcomes to standardize need (5, 27). Similarly, we classify population size (PS) as a “need” variable because it reflects absolute healthcare demand, complementing per-capita indicators like health outcomes (e.g., MMR) that measure intensity of need. While MMR shows maternal risk per 100,000 births, PS determines total required resources (e.g., a province of 10 M people needs more hospitals than one with 1 M, even if their MMRs are equal). Empirically, PS’s negative elastic coefficient in our model (Table 2) reveals systemic inequity: larger populations receive fewer per-capita resources. Policy-wise, this aligns with WHO guidelines and China’s health planning, where infrastructure quotas are population-based but adjusted for mortality rates. Furthermore, we incorporate population size (PS) as a variable of necessity to mitigate two significant allocation biases. Firstly, it helps to prevent the neglect of densely populated regions; for example, distributing equal per capita resources to both Shanghai and Tibet would not adequately reflect the considerably higher total service demand in Shanghai. Secondly, it addresses the potential misallocation of specialized resources: regions with high maternal mortality rates (MMR) necessitate a greater number of obstetric specialists, whereas regions with high population sizes (PS) require an increased number of general hospitals to accommodate their larger populations. This differentiation ensures that resources are appropriately aligned with both the magnitude and nature of healthcare needs across various regions.

**Table 2:** Healthcare resource allocation in 31 provinces (cities, autonomous regions) of China from 2009 to 2021.

Provinces (Cities, autonomous regions)	IPK				BPK				DPK				TPK				NPK			
	2009	2013	2017	2021	2009	2013	2017	2021	2009	2013	2017	2021	2009	2013	2017	2021	2009	2013	2017	2021
Beijing	0.55	0.46	0.46	0.49	5.13	4.92	5.56	5.95	4.70	5.85	4.10	5.14	12.92	15.46	11.30	13.20	4.95	6.36	4.80	5.67
Tianjin	0.35	0.32	0.36	0.44	3.77	3.92	4.39	5.00	2.59	3.18	2.50	3.77	6.90	8.05	6.50	8.87	2.34	2.95	2.50	3.41
Hebei	1.15	1.07	1.08	1.18	3.31	4.14	5.25	6.11	1.32	2.00	2.00	3.41	3.71	4.44	5.00	7.51	1.04	1.49	2.10	3.02
Shanxi	1.16	1.11	1.15	1.18	4.22	4.76	5.34	6.58	2.02	2.50	2.20	3.26	5.38	5.77	6.30	8.09	1.65	2.12	2.60	3.57
Neimenggu	0.92	0.93	0.96	1.04	3.56	4.81	5.94	6.94	2.48	2.52	2.40	3.51	5.50	6.01	7.10	8.82	1.44	2.12	2.80	3.71
Liaoning	0.80	0.81	0.82	0.78	4.41	5.51	6.83	7.67	1.98	2.44	2.40	3.12	5.32	6.01	6.70	7.90	1.99	2.44	2.90	3.61
Jilin	0.68	0.72	0.77	1.07	3.95	4.84	5.66	7.43	1.94	2.31	2.30	3.68	4.87	5.45	6.20	9.15	1.56	1.97	2.50	4.12
Heilongjiang	0.57	0.56	0.54	0.66	3.83	4.93	6.38	8.34	1.62	2.13	2.00	3.10	4.56	5.49	6.10	7.95	1.45	1.96	2.40	3.43
Shanghai	0.20	0.20	0.21	0.25	4.51	4.73	5.57	6.44	3.46	4.05	2.70	3.38	9.48	10.97	7.70	9.20	3.73	4.74	3.50	4.17
Jiangsu	0.39	0.39	0.40	0.43	3.21	4.64	5.84	6.45	1.50	2.23	2.30	3.21	4.16	5.63	6.80	8.13	1.50	2.29	3.00	3.63
Zhejiang	0.56	0.55	0.57	0.54	3.23	4.18	5.54	5.66	1.98	2.86	2.70	3.56	5.65	7.30	8.10	8.85	1.87	2.75	3.30	3.83
Anhui	0.40	0.41	0.39	0.48	2.85	3.91	4.89	6.72	0.94	1.42	1.60	2.82	3.07	3.66	5.00	7.12	1.03	1.49	2.20	3.29
Fujian	0.73	0.75	0.70	0.69	2.84	4.14	4.66	5.35	1.34	2.00	1.90	2.65	3.74	5.44	5.90	7.03	1.37	2.20	2.60	3.11
Jiangxi	0.77	0.86	0.82	0.81	2.60	3.85	5.06	6.80	1.09	1.46	1.50	2.47	3.25	3.94	5.10	6.77	1.16	1.62	2.30	3.10
Shandong	0.86	0.77	0.79	0.84	4.65	5.03	5.84	6.63	1.54	2.41	2.30	3.37	4.39	6.21	6.90	8.39	1.48	2.50	2.90	3.70
Henan	0.80	0.76	0.74	0.79	3.19	4.57	5.85	7.30	1.01	1.64	1.70	3.01	3.38	4.24	6.10	7.65	1.05	1.60	2.50	3.32
Hubei	0.57	0.61	0.62	0.63	3.27	4.97	6.37	7.44	1.34	1.90	2.10	2.91	4.02	5.01	6.80	7.83	1.42	2.07	3.10	3.68
Hunan	0.86	0.93	0.85	0.84	3.31	4.69	6.59	8.04	1.13	1.78	1.90	2.91	3.62	4.52	6.10	7.64	1.19	1.76	2.50	3.61
Guangdong	0.44	0.45	0.45	0.46	2.68	3.55	4.41	4.64	1.56	2.40	1.90	2.52	5.04	6.32	6.30	6.88	1.83	2.48	2.80	3.17
Guangxi	0.67	0.72	0.70	0.68	2.71	3.97	4.94	6.33	1.02	1.54	1.70	2.62	3.32	4.44	6.20	7.82	1.21	1.75	2.70	3.62
Hainan	0.54	0.56	0.56	0.62	2.72	3.59	4.53	6.02	1.24	1.84	1.90	2.91	4.30	5.29	6.50	7.89	1.73	2.30	3.10	3.77
Chongqing	0.58	0.64	0.64	0.67	3.24	4.96	6.71	7.50	1.00	1.64	1.80	2.87	3.05	4.23	6.20	7.68	0.97	1.65	2.80	3.55
Sichuan	0.89	0.99	0.97	0.96	3.36	5.26	6.79	7.91	1.21	1.90	2.00	2.99	3.37	4.68	6.40	8.04	1.01	1.74	2.80	3.66
Guizhou	0.70	0.83	0.78	0.76	2.76	4.76	6.51	7.71	0.81	1.31	1.70	2.74	2.37	3.64	6.30	8.03	0.78	1.37	2.70	3.68
Yunnan	0.49	0.52	0.51	0.57	3.07	4.48	5.72	7.04	1.12	1.63	1.60	2.68	3.02	4.20	5.90	8.12	1.04	1.59	2.70	3.89
Xizang	1.67	2.16	2.03	1.89	2.86	3.53	4.78	5.37	1.20	1.63	1.70	2.90	3.49	3.67	4.90	7.00	0.69	0.76	1.30	2.13
Shanxi	0.91	0.99	0.94	0.88	3.61	4.92	6.29	7.20	1.49	1.88	2.00	3.05	4.46	6.04	8.10	9.32	1.42	2.26	3.30	4.03
Gansu	0.99	1.03	1.10	1.03	3.19	4.49	5.58	7.36	1.13	1.65	1.70	2.84	3.38	4.33	5.60	8.07	0.98	1.50	2.20	3.68
Qinghai	1.07	1.04	1.07	1.08	3.45	5.11	6.41	7.10	1.58	2.31	2.20	3.16	4.43	5.66	7.00	8.70	1.44	2.01	2.80	3.59
Ningxia	0.66	0.65	0.63	0.63	3.54	4.76	5.84	5.68	1.70	2.14	2.40	3.11	4.48	5.58	7.30	8.36	1.55	2.09	3.20	3.76
Xinjiang	0.66	0.82	0.77	0.66	4.97	6.06	6.85	7.19	1.79	2.34	2.10	2.73	5.47	6.43	7.10	7.74	1.92	2.05	2.90	3.30
Average National level	0.73	0.76	0.75	0.78	3.48	4.58	5.71	6.71	1.64	2.22	2.11	3.11	4.65	5.75	6.56	8.19	1.57	2.19	2.77	3.61
Rise rate				6.85%				92.82%				89.63%				76.13%				129.94%

IPK, institutions per 1,000 people; BPK, beds per 1,000 people; DPK, doctors per 1,000 people; TPK, technicians per 1,000 people; NPK, nurses per 1,000 people.

#### 2.1.2 Data processing

The data processing involved three principal steps: (1) Data cleaning, which included cross-validation with provincial health bulletins, the correction of 23 outliers (representing 0.7% of the total) through the interquartile range (IQR) method, and the standardization of units across different years (for instance, ensuring a consistent classification of township hospital counts); (2) Addressing missing data, where the maternal mortality rate (MMR) for Tibet in 2015 was imputed using predictive mean matching and subsequently validated against trends from adjacent years, revealing less than a 5% fluctuation between 2014 and 2016. Additionally, 0.9% of missing values in the insured population (NI) were addressed through province-level linear interpolation; and (3) Quality control measures, which included consistency checks (for example, confirming that the number of reported beds did not exceed facility capacity) and external validation against the World Health Organization’s health system reports for China from 2019.

### 2.2 Methods

#### 2.2.1 Concentration index

The concentration index (CI) method (28, 29) assesses health equity across socioeconomic groups. This study used CI to evaluate healthcare resource allocation fairness (30, 31). Ranging from −1 (pro-poor) to +1 (pro-rich), with zero indicating perfect equity, CI’s absolute value reflects socioeconomic disparity magnitude. Widely adopted in health equity research (32), CI sensitively measures resource distribution inequalities. The formula for calculating the CI is as follows:


(1) $$C=\frac{2}{\mu }\mathrm{cov}(h,r).$$

In Equation 1, where C is concentration index; h is health index; r is the fractional rank of individual in the distribution of socio-economic position; *μ* is the mean of the healthcare resource allocation variable of the sample and cov denotes the covariance.

#### 2.2.2 Indirect standardized concentration index

The ISCI controls the non-need variables at the same level, uses multiple linear regression to calculate the expected amount of healthcare resources based on need variables that affect the amount of health resources, and then calculates the expected values of concentration index of health resource allocation. The difference between it and the total concentration index is the inequity caused by the economic level (27). The specific steps are as follows:

Firstly, establish the regression equation using the least squares method:


(2) $$y_{i}=\alpha +\sum_{i}\beta_{j}x_{\mathrm{ji}}+\sum_{k}\gamma_{k}z_{\mathrm{ki}}+\varepsilon_{i}.$$

In the Equation 2, i represents the individual, x_j_ represents the “need” variables for which we want to standardize; and **z**_k_ represents the non-need variables that for which we do not want to standardize but to control in the estimation of the β_j_.

Secondly, in the adjusted regression model, the non-need variables are used to predict the amount of healthcare resources determined by the need variables.


(3) $${\overset{\wedge }{y}}_{i}^{x}=\overset{\wedge }{\alpha }+\sum_{j}{\overset{\wedge }{\beta }}_{j}x_{\mathrm{ji}}+\sum {\overset{\wedge }{\gamma }}_{k}{\bar{z}}_{k}.$$

In the above Equation 3, we replace the “non-need” variables in the model with the sample mean to eliminate the influence of these variables and highlight the role of the “need” variables, resulting in the x-expected ${\hat{y_{i}}}^{x}$ amount of healthcare resources determined by the need variables.

Finally, using Equation 3 to abstain the indirectly need-standardize the amount of healthcare resources as the difference between actual amount of healthcare resources (y_i_) and ${\hat{y_{i}}}^{x}$ , plus the mean of predictions ( $\bar{y}$ ) were obtained:


(4) $${\overset{\wedge }{y}}_{i}^{\text{IS}}=y_{i}-{\overset{\wedge }{y}}_{i}^{x}+\bar{y}.$$

In the above Equation 4, ^−^y is the average amount of regional healthcare resources; ${\hat{y_{i}}}^{\text{IS}}$ the indirectly need- standardized amount of healthcare resources.

#### 2.2.3 Concentration index decomposition

The concentration index decomposition method separates the contributions of various influencing factors involved in the CI concentration index, and analyzes the regional differential influences of health resource allocation equity in China by studying the influence of “need” and “non-need” variables (such as socio-economic factors) of healthcare resource allocation. In this study, the dependent variables are indicators of health resource allocation: IPK, BPK, DPK, TPK, NPK. The independent variables are need variables of PS, PD, MMR, RBWL25, PMR, and GL. The non-need variables are GHE, OPP, PCHE, NI, DIUR, NIF, PCGDP, and UL.

The linear regression model established in this study is as follows:


(5) $$y_{i}=\alpha +\sum_{k}\beta_{k}x_{\mathrm{ki}}+\varepsilon_{i}.$$

The above Equation 5 is composed of the decomposition concentration index CI, where y_i_ is the dependent variable of healthcare resource allocation, i is the individual, k is the determinant, *α* is the intercept, *β*_k_ is the regression coefficient, which represents the marginal impact of the corresponding factor on healthcare resource allocation, x_ki_ is the relative contribution of individual i to the determinant k on healthcare resource allocation, and *ε*_i_ is the interference term. Assuming that individuals all face the same coefficient vector, the differences in income-based y between individuals can be derived from the differences in various determinants. So the concentration index can be decomposed into as follows:


(6) $$C=\sum_{k}(\frac{\beta_{k}\bar{x_{k}}}{\mu })C_{k}+\frac{{\mathrm{GC}}_{\varepsilon }}{\mu }.$$

The above Equation 6 shows that the overall inequality in healthcare resource allocation has two components, a deterministic or “explained” component and an “unexplained “component. In the former component βk is the coefficient from a regression of healthcare resource allocation variable on determinant k, $\bar{x_{k}}$ is the mean of determinant k, *μ* is the mean of the healthcare resource allocation index, and Ck is the CI for determinant k. In the latter component, GCε is the generalized CI for the error term. The CI indicates that the concentration index of healthcare resource allocation is equal to the weighted concentration index of the “need” and “non-need” variables (the weight is the mean of the variable * the quotient of its marginal effect and the mean of y, which is the corresponding elasticity coefficient), and the product of the concentration index of each “need” and “non-need” variable and its weighted concentration index is the contribution to the inequality of healthcare resource allocation. Calculate the contribution rate of each variable based on the ratio of the concentration index of the “need” and “non-need” variables to the overall concentration index. Since the overall concentration index is equivalent to the algebraic sum of the concentration indices of each variable, and the concentration index can be positive or negative, the contribution rate of a single variable can be less than or greater than 100%.

However, the traditional contribution rate formula encounters a significant limitation when the Concentration Index (CI) equals zero, resulting in a zero denominator and yielding nonsensical outcomes, such as values exceeding 1,000% or other anomalous figures. To address this issue, we propose an alternative calculation for the contribution rate (CR) that utilizes the sum of the absolute values of each factor’s contribution as the denominator. The updated formula is present as follows:


(7) $$\mathrm{CR}=\frac{|{\beta_{k}}^{*}\bar{x_{k}}*C_{k}|}{\sum |{\beta_{k}}^{*}\bar{x_{k}}*C_{k}|}\times 100\%.$$

The symbolic expressions in the above Equation 7 follow the same conventions as Equation 6. This approach enhances the robustness and interpretability of the results in the following ways: (1) Mathematical Robustness. This formulation effectively circumvents the problem of a zero denominator that arises when the CI is equal to zero. (2) Interpretability: The sum of the contribution rates across all variables is constrained to equal 100%, facilitating clearer understanding of the results. (3) Direction Preservation: The positive or negative sign of the coefficient (βk) continues to provide insight into whether a particular factor promotes or inhibits equity.

It can be seen that the concentration index of healthcare resource allocation can be decomposed into the weighted sum of concentration indices of various explanatory factors, and the weight is the elasticity of healthcare resource allocation with respect to factor *k*. The residual term reflects the unequal allocation of healthcare resources that is not explained by the independent variables. If the model is set properly, the contribution of the residual term should be close to zero.

## 3 Results

### 3.1 Analysis of descriptive statistics for the variables of interest

Analysis of descriptive statistics for 20 variables across 279 observations include mean values such as IPK (0.75), BPK (4.57), and PCGDP (45,716.68 CNY), with standard deviations indicating variability (e.g., PD: 7,013.3). Data quality is verified through external sources (e.g., WHO, NHC), showing high consistency (98% match for IPK). Skewness is suggested by wide ranges (e.g., PD: 34.46–40,477.74). Monetary variables (e.g., DIUR) and health metrics (e.g., MMR: 20.46) align with national reports. See detail in Supplementary Table S1.

### 3.2 Analysis of health resource allocation across 31 provincial regions of China from 2009 to 2021

Table 2 illustrates that from 2009 to 2021, there has been a notable increase in the indices of IPK, BPK, DPK, TPK, and NPK in China. The most significant growth was observed in NPK, which experienced an increase of 129.94%, followed by BPK at 92.82%, DPK at 89.63%, and TPK at 76.13%. In contrast, IPK exhibited only a marginal increase of 6.85% (refer to Table 2).

**Figure 1:**
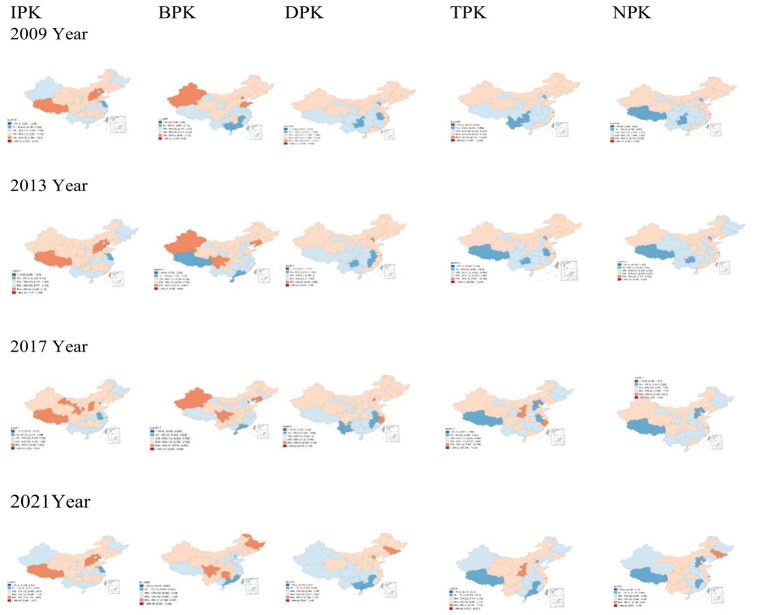
Spatial trends of distribution of healthcare resource allocation in provinces (cities, autonomous regions) of mainland China from 2009 to 2021. IPK, institutions per 1,000 people; BPK, beds per 1,000 people; DPK, doctors per 1,000 people; TPK, technicians per 1,000 people; NPK, nurses per 1,000 people.

An analysis of the geographic spatial distribution trends over the 13-year period, as depicted in Figure 1, reveals considerable regional disparities in the distribution of healthcare resources. The most prominent patterns identified include: (1) A persistent concentration of human resources (DPK, TPK, NPK) in the eastern regions, despite a slight westward expansion of TPK; (2) A discernible trend of redistribution for BPK from eastern provinces, such as Shandong and Jiangsu, which have experienced declines, to central and western regions, although Xinjiang and Ningxia have consistently remained at low levels; (3) The allocation of IPK exhibited a complex pattern, with western provinces like Shanxi and Yunnan maintaining low levels, while central and western regions overall received the majority of allocations, and certain eastern provinces, such as Hebei, consistently received high allocations throughout the period. Notably, NDK demonstrated a relatively uniform distribution across all regions, with a slight concentration in the eastern areas.

### 3.3 Analysis of CI and ISCI of healthcare resource allocation across various provincial regions of China from 2009 to 2021

Table 3 reveals three significant patterns in the allocation of healthcare resources. Firstly, the consistently negative concentration index (CI) for the Index of Public Knowledge (IPK) from 2009 to 2021 indicates a disproportionate allocation of institutional resources favoring economically disadvantaged areas, which is consistent with prior research. Secondly, the Budget for Public Knowledge (BPK) exhibits a notable reversal; it initially favored developed regions (as indicated by a positive CI from 2009 to 2012) before transitioning to support underdeveloped areas (reflected in a negative CI from 2013 to 2021, with the exception of 2014). Most importantly, health workforce resources, including the Distribution of Health Personnel (DPK), the Distribution of Medical Equipment (TPK), and the Distribution of Health Facilities (NPK), consistently maintained positive CIs throughout the study period, indicating a persistent bias toward developed regions.

**Table 3:** CI and ISCI of healthcare resource allocation across various provincial regions of China from 2009 to 2021.

Year	IPK		BPK		DPK		TPK		NPK	
	CI	ISCI	CI	ISCI	CI	ISCI	CI	ISCI	CI	ISCI
2009	−0.053	−0.077	0.035	0.102	0.148	0.147	0.131	0.145	0.151	0.100
2010	−0.072	−0.074	0.028	0.103	0.148	0.163	0.135	0.165	0.154	0.145
2011	−0.076	−0.079	0.077	0.155	0.129	0.137	0.133	0.155	0.147	0.135
2012	−0.076	−0.077	0.007	0.075	0.088	0.096	0.066	0.088	0.073	0.066
2013	−0.067	−0.059	−0.002	0.067	0.126	0.136	0.124	0.147	0.132	0.133
2014	−0.036	−0.028	0.001	0.067	0.058	0.069	0.054	0.080	0.063	0.065
2015	−0.084	−0.072	−0.003	0.063	0.057	0.070	0.052	0.079	0.018	0.020
2016	−0.083	−0.056	−0.007	0.063	0.067	0.090	0.050	0.080	0.058	0.070
2017	−0.085	−0.051	−0.015	0.055	0.070	0.093	0.047	0.077	0.050	0.065
2018	−0.084	−0.039	−0.014	0.056	0.070	0.097	0.050	0.082	0.051	0.073
2019	−0.106	−0.048	−0.021	0.048	0.041	0.067	0.038	0.069	0.045	0.070
2020	−0.118	−0.058	−0.035	0.030	0.021	0.046	0.017	0.047	0.019	0.044
2021	−0.111	−0.049	−0.042	0.022	0.017	0.040	0.012	0.042	0.013	0.034

CI, concentration index; ISCI, indirect standardization concentration Index. IPK, institutions per 1,000 people; BPK, beds per 1,000 people; DPK, doctors per 1,000 people; TPK, technicians per 1,000 people; NPK, nurses per 1,000 people.

The analysis of distribution equity reveals two critical findings: while inequality in IPK has intensified (as evidenced by increasing absolute CI values), other resources have shown gradual improvement, as indicated by decreasing CIs for BPK, DPK, TPK, and NPK. This trend suggests progress toward a more equitable distribution, particularly concerning hospital beds and medical personnel. The CI values for 2021 (−0.042 for BPK, 0.017 for DPK, 0.012 for TPK, and 0.013 for NPK) illustrate this emerging equilibrium. Notably, the trends in the Index of Socioeconomic Concentration Index (ISCI) align perfectly with these CI patterns throughout the period from 2009 to 2021.

### 3.4 Analysis of contribution factors to the CI of healthcare resource allocation in China from 2009 to 2021

**Figure 2:**
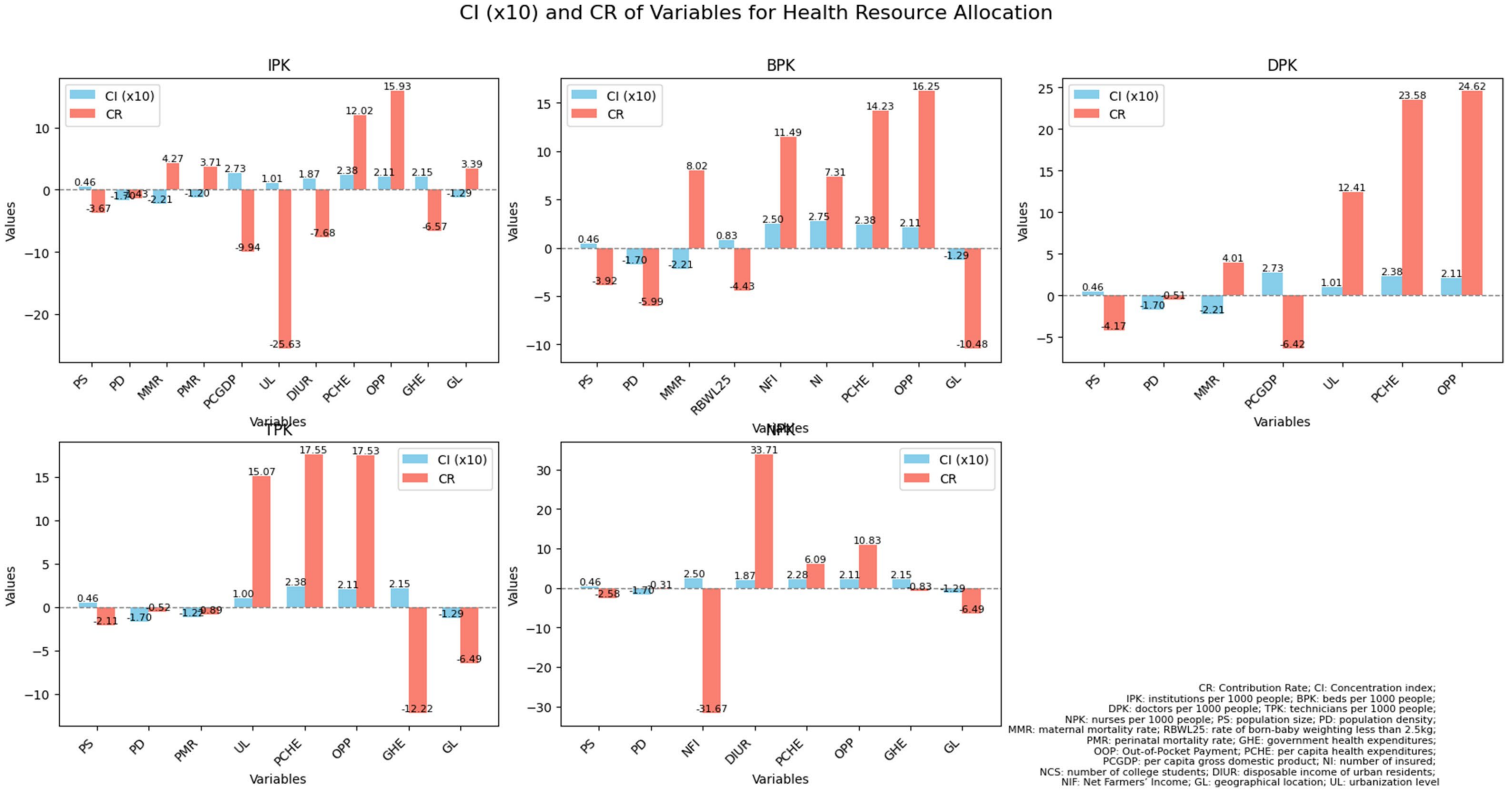
An analysis of contribution rates and concentration indices pertaining to health resource allocation in China from 2009 to 2021. CR, contribution rate; CI, concentration index; IPK, institutions per 1,000 people; BPK, beds per 1,000 people; DPK, doctors per 1,000 people; TPK, technicians per 1,000 people; NPK, nurses per 1,000 people; PS, population size; PD, population density; MMR, maternal mortality rate; RBWL25, rate of born-baby weighting less than 2.5 kg; PMR, perinatal mortality rate; GHE, government health expenditures; OOP, Out-of-Pocket Payment; PCHE, per capita health expenditures; PCGDP, per capita gross domestic product; NI, number of insured; NCS, number of college students; DIUR, disposable income of urban residents; NFI, Net Farmers’ Income; GL, geographical location; UL, urbanization level.

As indicated in Table 4 and Figure 2, the “need” variables—PS, PD and MMR—were significant factors in the unequal distribution of healthcare resources. The contribution rates of PS to the disparity in healthcare resources, specifically the numbers of IPK, BPK, DPK, TPK, and DPK, were −3.67, −3.92%,- 4.17, −2.11%, and −2.58%, respectively. For PD, the rates were −1.43, −5.99%, −0.51, −0.52%, and −0.31%. MMR’s contributions were 4.27, 8.02, 4.01, 5.09, and 2.77%. Notably, RBWL25 had a significant impact on the unfair distribution of beds, contributing −4.43%, while PMR had a stronger effect on the distribution of institutions and nurses, with contributions of 3.71 and 2.27%, respectively. Regarding CI, both PS and RBWL25 had positive indices for healthcare resources, indicating that these factors favored resource allocation in economically developed areas. In contrast, PD, GL, and MMR showed negative values, suggesting they favored resource distribution in less economically developed regions.

**Table 4:** Decomposition of concentration index of health resource allocation in China from 2009 to 2021.

Determinants	IPK			BPK			DPK			TPK			NPK		
	EC	CI	CR (%)	EC	CI	CR (%)	EC	CI	CR (%)	EC	CI	CR (%)	EC	CI	CR (%)
Need variables															
PS	−0.368	0.046***	−3.667	−0.247	0.046***	−3.920	−0.252	0.046***	−4.172	−0.129	0.046**	−2.107	−0.352	0.046***	−2.581
PD	0.039	−0.170***	−1.434	0.010	−0.170**	−5.985	0.008	−0.170**	−0.507	0.009	−0.170***	−0.520	0.011	−0.170*	−0.309
MMR	−0.090	−0.221**	4.274	−0.106	−0.221***	8.019	−0.051	−0.221*	4.013	−0.065	−0.221	5.090	−0.079	−0.221	2.767
RBWL25	−0.072	0.083	−1.286	−0.157	0.083***	−4.432	−0.018	0.083	−0.528	0.004	0.083	0.104	−0.012	0.083	0.160
PMR	−0.140	−0.122*	3.713	0.000	−0.122	−0.002	0.005	−0.122	−0.214	0.021	−0.122*	−0.889	−0.117	−0.122	2.271
Non-need variables															
PCGDP	−0.168	0.273*	−9.938	−0.118	0.273	−11.017	−0.066	0.273**	−6.421	−0.057	0.273	−5.472	−0.021	0.273	−0.891
UL	−1.180	0.101***	−25.632	−0.059	0.101	−2.030	0.345	0.101*	12.415	0.425	0.100**	15.074	0.242	0.101	3.863
NFI	−0.023	0.250	−1.265	0.134	0.250*	11.491	0.004	0.250	0.341	−0.022	0.250	−1.923	−0.799	0.250***	−31.670
DIUR	−0.191	0.187*	−7.685	0.056	0.187	3.597	0.041	0.187	2.766	0.158	0.187	10.396	1.137	0.187***	33.708
NCS	0.003	0.082	0.050	−0.028	0.082	−0.801	0.035	0.082	1.040	0.067	0.082	1.932	0.075	0.082	0.984
NI	0.053	0.275	3.142	0.077	0.275*	7.308	0.043	0.275	4.243	0.028	0.275	2.710	−0.035	0.275	−1.549
PCHE	0.233	0.238***	12.020	0.174	0.238***	14.235	0.276	0.238***	23.578	0.209	0.238**	17.546	0.161	0.228*	6.089
OPP	0.350	0.211***	15.934	0.225	0.211**	16.251	0.327	0.211***	24.618	0.236	0.211***	17.533	0.324	0.211**	10.828
GHE	−0.141	0.215*	−6.565	−0.079	0.215	−5.816	−0.189	0.215	−14.501	−0.161	0.215***	−12.217	−0.024	0.215**	−0.828
GL	−0.122	−0.129*	3.394	0.238	−0.129***	−10.482	0.014	−0.129	−0.641	0.143	−0.129***	−6.486	0.074	−0.129	−1.502
*F* value	54.06			68.49			64.49			105.84			68.15		
*p* value	<0.001			<0.001			<0.001			<0.001			<0.001		
R^2^	0.4341			0.6671			0.7152			0.7752			0.6136		

The elasticity coefficient(EC) is the weight obtained when decomposing the concentration index, which is the mean of each factor multiplied by its marginal effect divided by the mean of y. CR, Standard Contribution Rate, calculated as the percentage share of each factor’s absolute value (totaling 100%), where the sign indicates directional impact—positive values favor resource allocation to higher-income areas, negative values the reverse;*indicates *p <* 0.05, **indicates *p* < 0.01, ***indicating *p* < 0.001; CI, Concentration index; IPK, institutions per 1,000 people; BPK, beds per 1,000 people; DPK, doctors per 1,000 people; TPK, technicians per 1,000 people; NPK, nurses per 1,000 people; PS, population size; PD, population density; MMR, maternal mortality rate; RBWL25, rate of born-baby weighting less than 2.5 kg; PMR, perinatal mortality rate; GHE, government health expenditures; OOP, Out-of-Pocket Payment; PCHE, per capita health expenditures; PCGDP, per capita gross domestic product; NI, number of insured; NCS, number of college students; DIUR, disposable income of urban residents; NIF, Net Farmers’ Income; GL, geographical location; UL, urbanization level.

The impact of these key factors on the unfair distribution of health resources varies. For example, the contribution rates of PS, PD, MMR, RB2L25 and PMR to the unequal distribution of institutions were −3.67, −1.43%, 4.27,-1.29 and 3.71%, respectively. This shows that PS, PD and RB2L25 reduced the unfairness by over 1–4%, while MMR, and PMR helped increased it by 3–4%. In terms of bed distribution, the contribution rates were −3.92, −5.99%, 8.02,-4.43% indicating that PS, PD and low birth weight alleviated unfairness by varying degrees of 3–6%, while MMR worsened it by over 8%. Similarly, PS, PD, and PMR contributed to reducing the unfair distribution of health human resources, whereas MMR aggravated the issue by more than 4%% for doctors.

According to Table 4 and Figure 2, among the “non-need” variables, GL, UL, OPP, PCHE, PCGDP, DIUR, GHE, and NI are the main contributing factors, and the concentration index is all positive except for GL, indicating that the later seven factors promote the allocation of health institutions, beds, and health personnel to economically developed provinces, while GL to economically undeveloped ones. The contribution rates of government health expenditure to the unequal allocation of institutions, technicians, and nurses are −6.57, −12.22%, and −0.83%, respectively; The contribution rates of personal health expenditure to the unequal allocation of institutions, beds, doctors, medical technicians, and nurses are 15.93, 16.25, 24.62, 17.53, and 10.83%, respectively; The contribution rates of per capita health expenditure to the unequal allocation of institutions, beds, doctors, technicians, and nurses are 12.02, 14.24, 23.58, 17.55, and 6.09%, respectively; The contribution rates of the number of insured individuals to the unfair allocation of beds, are 7.31%; The contribution rates of DIUR areas to the unequal allocation of institutions, and nurses are −7.69 and 33.71%, respectively; The contribution rates of UL to the unequal allocation of institutions, doctors, and technicians are −25.63, 12.42 and 15.07%, respectively. The contribution rates of PCGDP to the unequal allocation of institutions and doctors are −9.94% and −6.42%, respectively. The above factors all lead to a bias in the allocation of health resources toward economically developed provinces. It is worth noting that the contribution rate of NFI to the unfair allocation of beds and nurses is also relatively high, at 11.49% and −31.67% respectively, which promotes the resource allocation of these two areas to be biased toward economically developed regions. In contrast, GL accounted for 3.39, −10.48%, and −6.49% of the disparities in the distribution of healthcare institutions, beds, and technicians, respectively, with all CIs indicating negative values for these three categories of healthcare resources. This suggests that GL facilitates the allocation of healthcare institutions, beds, and personnel to provinces that are economically disadvantaged.

## 4 Discussion

### 4.1 Structural imbalances in healthcare resource distribution: infrastructure vs. human resources

China has experienced an increase in the number of healthcare professionals, medical institutions, hospital beds, and nursing staff; however, this growth is accompanied by notable regional disparities. Specifically, while medical institutions and hospital beds are being relocated to the less developed central and western regions, healthcare personnel continue to be predominantly concentrated in the eastern regions (5, 15, 28–33). These disparities can be attributed to variations in economic conditions, social factors, and levels of investment. Following the medical reforms initiated in 2009, various western development initiatives, such as the Western Health Talent Training Project and infrastructure investments in regions like Xinjiang and Tibet, have contributed to a more equitable distribution of healthcare facilities. Nevertheless, the human resources sector is experiencing a “Matthew effect,” as healthcare workers tend to migrate back to the eastern regions due to inadequate compensation (34), ineffective policies (35), unsustainable incentives (34, 36), limited career opportunities (37), and the allure of talent in the east (38). In order to mitigate the deficit of healthcare human resources in central and western China, several strategic measures are recommended: (1) enhancing compensation frameworks and granting local governments greater autonomy in the areas of staffing and recruitment; (2) optimizing the doctor-to-nurse ratio to align with the World Health Organization’s standard of 1:2; (3) improving targeted training initiatives by offering competitive salaries, benefits, and career advancement opportunities to encourage graduates to remain in underserved regions; and (4) reinforcing the support for eastern-western regional partnerships through the implementation of comprehensive evaluation systems that connect the performance of medical personnel in these initiatives to promotional opportunities, thereby ensuring a sustainable and equitable distribution of healthcare workers.

### 4.2 Need variables’ dual role: alleviating vs. exacerbating healthcare disparities across regions

Factors alleviating unequal healthcare resource distribution include PS, PD, and RBWL25 (particularly for beds and doctors), while MMR (most impactful) and PMR (institutions and nurses) exacerbate disparities. Economically developed regions benefit more from PS and RBWL25, whereas less developed areas rely more on PD and MMR-related resource allocation. This phenomenon can be attributed to the fact that developed regions generally possess more robust infrastructure (PS) and superior neonatal care (RBWL25), which facilitates a more effective distribution of resources (39, 40). In contrast, less developed regions rely on population density (PD) to achieve basic healthcare coverage and exhibit elevated maternal mortality rates (MMR), indicative of systemic deficiencies in healthcare access. The observed PMR effect arises from an urban bias in institutional investments and the allocation of nursing personnel. In essence, affluent areas capitalize on systemic advantages to enhance resource optimization, whereas economically disadvantaged regions are hindered by demographic factors and mortality-driven resource allocation patterns that sustain existing inequities. To mitigate disparities in healthcare resources, policymakers should consider the following strategies: (1) Establish targeted funding mechanisms aimed at reallocating resources from developed areas to underserved regions, with a particular emphasis on interventions related to maternal and child health (MMR/RBWL25); (2) Create allocation models that adjust for population density (PD) to ensure an equitable distribution of hospital beds and medical professionals in rural settings; (3) Initiate urban–rural partnership programs that facilitate the sharing of resources and personnel between advanced hospitals and underserved healthcare institutions; (4) Implement conditional fiscal transfers that incentivize provinces to address disparities in the distribution of nursing staff related to PMR; (5) Develop a national healthcare resource monitoring system equipped with real-time alerts for disparities. These initiatives should be integrated with economic development programs in disadvantaged areas to progressively diminish reliance on need-based resource allocation.

### 4.3 Non-need vs. economic factors: divergent impacts on health equity

Healthcare resource allocation is predominantly influenced by economic factors, with personal expenditures leading to a concentration of resources in more developed regions. Although government spending and economic development efforts can mitigate some disparities, they do not eliminate regional imbalances. Urbanization tends to enhance the distribution of healthcare institutions, yet it exacerbates the allocation of healthcare professionals. Additionally, geographic factors can provide advantages to underserved areas. The identified disparities arise from inherent structural imbalances within healthcare systems. Affluent regions tend to amass greater resources as a result of elevated personal expenditure capabilities and superior infrastructure, while governmental redistribution initiatives are inadequate to counteract these market dynamics (41–43). The number of insured contributes to unfair bed distribution (7.31%) by disproportionately favoring developed regions through higher coverage rates, larger insurance pools, and urban-biased reimbursement policies, despite post-2013 redistribution efforts to disadvantaged areas as shown in Table 4.

Urbanization leads to a concentration of specialized medical professionals, whereas geographical considerations can be mitigated through policy interventions in less accessible areas. To address healthcare disparities, it is essential for the government to implement strategies that do not solely rely on need-based factors. First, the introduction of progressive taxation policies for healthcare financing is crucial to counteract the concentration of resources in affluent regions, which is often exacerbated by market dynamics. This strategy aims to redistribute financial resources from high-income areas to underserved populations while maintaining high standards of healthcare quality. Second, the establishment of mandatory rotation programs for healthcare professionals between urban and rural environments is necessary to combat the clustering of medical professionals in metropolitan areas. By offering incentives such as career advancement opportunities and financial compensation, this initiative seeks to motivate specialists to practice in remote locations, thereby fostering a more equitable distribution of medical expertise. Third, the government will implement a comprehensive strategy that includes increasing insurance subsidies in underserved areas, adopting value-based payment models to prevent bed overconcentration, enhancing insurance portability for cross-regional care access, developing needs-based bed allocation policies, and prioritizing infrastructure investments in regions with low insurance coverage, thereby ensuring balanced and efficient healthcare service delivery nationwide. Fourth, the development of geographically weighted funding formulas is vital to prioritize investments in healthcare infrastructure in hard-to-reach areas. Funding allocations should be determined based on indices of remoteness and the specific health needs of populations, rather than relying exclusively on economic indicators, thus leveraging geographic advantages to improve access to healthcare services.

## 5 Conclusion

The research indicated that between 2009 and 2021, China experienced substantial advancements in healthcare resources; however, enduring regional disparities highlighted underlying structural imbalances. Although investments in infrastructure enhanced access in less developed areas, the distribution of human resources remained predominantly concentrated in the eastern regions, attributable to economic advantages, ineffective retention strategies, and trends in professional migration. The variables related to need exhibited a dual impact—specifically, the variables PS, PD, and RBWL25 mitigated disparities in the availability of hospital beds and physicians, whereas MMR and PMR exacerbated inequalities in maternal healthcare and nursing distribution. Concurrently, non-need economic factors, such as personal expenditures numbers of insured and urbanization, further exacerbated the concentration of resources in affluent regions, despite efforts aimed at geographic and fiscal redistribution. To promote equity, it is imperative for China to (1) reform compensation and autonomy policies to enhance the retention of medical professionals in western regions, (2) adjust funding models to prioritize allocations based on need and geographic considerations rather than solely on economic indicators, and (3) implement urban–rural partnerships that incorporate performance-based incentives. Furthermore, targeted investments in maternal health and the establishment of real-time monitoring systems for disparities are essential to mitigate market-driven inequities. These strategies provide a framework for developing nations facing analogous challenges in achieving equitable healthcare amidst rapid growth.

## Data Availability

The raw data supporting the conclusions of this article will be made available by the authors, without undue reservation.

## Glossary

CI: concentration index
ISCI: indirect standardized concentration index
IPK: institutions per 1,000 people
BPK: beds per 1,000 people
DPK: doctors per 1,000 people
TPK: technicians per 1,000 people
NPK: nurses per 1,000 people
PS: population size
PD: population density
MMR: maternal mortality rate
RBWL25: rate of born-baby weighting less than 2.5 kg
PMR: perinatal mortality rate
GHE: government health expenditures
OOP: out-of-pocket payment
PCHE: per capita health expenditures
PCGDP: per capita gross domestic product
NI: number of insured
NCS: number of college students
DIUR: disposable income of urban residents
NFI: net farmers’ income
GL: geographical location
UL: urbanization level
