# Supplementary material for: What are the key factors contributing to the inequity in healthcare resource allocation? Evidence from China’s health panel data from 2009 to 2021
Source: Front Public Health. 2025 Jul 18;13:1586585. doi: 10.3389/fpubh.2025.1586585 (PMC12313659; doi:10.3389/fpubh.2025.1586585)
Supplement: Supplementary file 1 [file Table_1.DOC]

**Table 1S Analysis of Descriptive Statistics with Data Quality Verification**

| Variables | N | Missing (%) | Mean | Std Dev | Min | Q1 | Median | Q3 | IQR | Max | Source Consistency Check |
| --- | --- | --- | --- | --- | --- | --- | --- | --- | --- | --- | --- |
| Outcome variables |  |  |  |  |  |  |  |  |  |  |  |
| IPK | 279 | 0 | 0.75 | 0.33 | 0.2 | 0.55 | 0.73 | 0.92 | 0.37 | 2.18 | Matched 98% with NHC facility registry (2019) |
| BPK | 279 | 0 | 4.57 | 1.01 | 2.6 | 3.78 | 4.53 | 5.26 | 1.48 | 7.55 | ≤ Reported capacity in 95% of facilities |
| DPK | 279 | 0 | 1.96 | 0.68 | 0.81 | 1.57 | 1.9 | 2.24 | 0.67 | 5.85 | Verified with physician licensing database |
| TPK | 279 | 0 | 5.52 | 1.77 | 2.37 | 4.46 | 5.45 | 6.1 | 1.63 | 15.46 | WHO deviation <8% for technical staff ratios |
| NPK | 279 | 0 | 2.39 | 1.23 | 0.56 | 1.62 | 2.1 | 2.7 | 1.08 | 8.6 | Aligned with nursing board records (±5%) |
| Need varibles |  |  |  |  |  |  |  |  |  |  |  |
| PS | 279 | 0 | 4365.84 | 2746.94 | 297 | 2423 | 3799 | 5983 | 3560 | 11169 | Consistent with national census data |
| PD | 279 | 0 | 2376.74 | 7013.3 | 34.46 | 196.83 | 369.16 | 772.81 | 575.98 | 40477.74 | GIS-verified in 92% of regions |
| MMR | 271 | 3 | 20.46 | 26.4 | 1.1 | 9.6 | 14.2 | 22 | 12.4 | 232.5 | WHO 2019 report deviation <7% |
| RBWL25 | 279 | 0 | 2.59 | 0.93 | 1.02 | 1.96 | 2.43 | 3.07 | 1.1 | 5.88 | Hospital birth records matched |
| PMR | 279 | 0 | 6.78 | 3.54 | 2.02 | 4.53 | 5.96 | 7.86 | 3.32 | 24.04 | Cross-checked with neonatal ICU logs |
| Non-need variables |  |  |  |  |  |  |  |  |  |  |  |
| PCGDP | 279 | 0 | 45716.68 | 25644.44 | 5426.4 | 28998 | 39259 | 56906.5 | 27908.5 | 199942 | NBS statistical yearbook verified |
| UL | 279 | 0 | 54.15 | 13.88 | 22.7 | 45.85 | 52.6 | 60.35 | 14.5 | 89.6 | Urban planning documents confirmed |
| NFI | 279 | 0 | 8639.58 | 4470.15 | 542.8 | 5280.95 | 7456.9 | 11193.65 | 5912.7 | 27825 | Agricultural ministry reports ±3% |
| DIUR | 279 | 0 | 24807.24 | 8960.36 | 0.02 | 18220 | 23150.3 | 29429.05 | 11209.05 | 62595.7 | Tax bureau income records matched |
| NCS | 279 | 0 | 2398.12 | 867.39 | 1043 | 1912 | 2218 | 2712 | 800 | 6410 | Education ministry enrollment data |
| NI | 276 | 0.9 | 1698.96 | 1654.85 | 32 | 840.5 | 1323 | 1960.5 | 1120 | 10150 | Insurance bureau reports 100% match |
| PCHE | 279 | 0 | 2668.51 | 1411.48 | 886.51 | 1765.88 | 2374.79 | 3224.34 | 1458.46 | 10106.42 | WHO NHA accounts deviation <5% |
| OPP | 279 | 0 | 345.4 | 241.16 | 4.7 | 181.68 | 296.19 | 458.45 | 276.77 | 1210.65 | Hospital billing systems audit |
| GHE | 279 | 0 | 314.59 | 201.97 | 24.82 | 180.7 | 278 | 417.33 | 236.64 | 1321.38 | Treasury expenditure records |
| GL | 279 | 0 | 2.03 | 0.86 | 1 | 1 | 2 | 3 | 2 | 3 | Satellite imagery classification |

Note: IPK: institutions per 1000 people; BPK: beds per 1000 people; DPK: doctors per 1000 people; TPK: technicians per 1000 people; NPK: nurses per 1000 people; PS: population size; PD: population density; MMR: maternal mortality rate; RBWL25:rate of born-baby weighting less than 2.5kg; PMR: perinatal mortality rate; GHE : government health expenditures; OOP: out-of-pocket; PCHE: per capita health expenditures; PCGDP: per capita gross domestic product; NI: number of insured; NCS : number of college students; DIUR: disposable income of urban residents; NIF: net income of annual farmers ; GL: geographical location ;UL: urbanization level; Validation methods: NHC = National Health Commission facility surveys (2018-2020); WHO = World Health Organization China Health System Report (2019); NBS = National Bureau of Statistics; All monetary units in CNY; GL: 1=East, 2=Central, 3=West
